# A Case of a New Ectoparasitic Fungal Infection of the Ant *Lasius distinguendus* (Hymenoptera, Formicidae) with Description of *Xenomorphomyces formicartus* gen. et sp. nov. (Capnodiales, Readerielliopsidaceae)

**DOI:** 10.3390/insects17080853

**Published:** 2026-08-16

**Authors:** Vadim Iliushin, Dmitry Shevchenko, Irina Kirtsideli, Dmitry Dubovikoff, Anna Kiyashko, Evgeny Abakumov

**Affiliations:** 1 Komarov Botanical Institute of the Russian Academy of Sciences, Professora Popova Street, 2, St. Petersburg 197022, Russia; ilva94@yandex.ru (V.I.); microfungi@mail.ru (I.K.); anna.kiyashko@binran.ru (A.K.); 2 D.I. Ivanovsky Academy of Biology and Medicine, Southern Federal University, Stachki Avenue, 194/1, Rostov-on-Don 344090, Russia; cheff7627d@gmail.com; 3 Federal Research Centre the Southern Scientific Centre of the Russian Academy of Sciences, Chekhov Avenue, 41, Rostov-on-Don 344006, Russia; 4 Department of Applied Ecology, Faculty of Biology, St. Petersburg State University, 16-Line of Vasilevskiy Island, 29, St. Petersburg 199178, Russia; e.abakumov@spbu.ru

**Keywords:** ants, Capnodiales, Lasius, microfungi, multi-gene phylogeny, myrmecopathogenic fungi, parasitic fungi, Readerielliopsidaceae

## Abstract

This study presents the first case of an ectoparasitic fungal infection of ants from southern Russia with the description of a new species *Xenomorphomyces formicartus* V.A. Iliushin & I.Y. Kirtsideli, **sp. nov.** of a new genus *Xenomorphomyces* **gen. nov.** belonging to the family Readerielliopsidaceae. The new species was found on *Lasius distinguendus* worker ants within a single nest in the Botanical Garden of the Southern Federal University, Rostov-on-Don, Rostov Region, Russia. The paper evaluates the phylogenetic position of the parasitic fungus within the Readerielliopsidaceae family based on the morphological observations and multilocus phylogenetic analysis. The family Readerielliopsidaceae belongs to the Capnodiales order, the class Dothideomycetes. Partial sequences of the loci encoding ribosomal polymerase II second-largest subunit (*RPB2*), 28S rDNA (LSU) and internal transcribed spacer rDNA region (ITS1-5.8S-ITS2) were analyzed. The asexual morph of *X. formicartus* was described. A comprehensive analysis of pathogenicity was performed using live ants, histological and micro-CT studies. The sequence data, as well as macro- and micromorphological characteristics, distinguish *X. formicartus* from all known species in the Readerielliopsidaceae.

## Simple Summary

*Xenomorphomyces formicartus* sp. nov.—a new fungus parasitic on the ant *Lasius distinguendus* (Emery, 1916)—is described from specimens collected at the Botanical Garden of the Southern Federal University (Rostov-on-Don, Russia). The new genus *Xenomorphomyces* is assigned to the family Readerielliopsidaceae on the basis of morphological and multilocus phylogenetic data. We also provide a description of its asexual morph. The combination of sequence and morphological data allows unambiguous separation of *X. formicartus* from its congeners within the family. Furthermore, histology and micro-CT reveal the pattern of pathogenesis within the host ant.

## 1. Introduction

Ants (Hymenoptera, Formicidae), as social insects, present a convenient target for a wide range of parasitic microorganisms, including fungi, due to their group lifestyle and frequent interactions between individuals, which increase opportunities for pathogen transmission [1,2,3,4,5]. However, fungal epidemics affecting entire ant colonies are rare. Most known cases involve the infection of individual ants, which can be attributed to the ants’ sophisticated behavioral and immunological defense mechanisms against pathogens [1,6]. Still, large-scale epizootics in ant colonies have been documented in some cases [7,8].

Depending on their negative effect on the host insect, these fungi can be classified as pathogenic (necrotrophs or hemibiotrophs, host-killing, such as *Beauveria*, *Conidiobolus*, *Metarhizium*, *Pandora*) or parasitic (depending on the living host, non-lethal, e.g., *Aegeritella*, *Hormiscium*, *Laboulbenia*, *Rickia*) [6,9,10,11]. They first infect the host via penetration of its cuticle and generally form special structures such as germ tubes, appressoria and/or penetration pegs [10]. Ectoparasites exhibit a range of contact modes with their hosts from merely attaching to the integuments to invasion into the body cavity via more or less pronounced penetration structures. Some species can invade the cuticle only superficially. It is considered that in this process the fungus obtains nutrients by absorption through the cuticle or via contact with underlying living tissues [12]. The harmful impact of ectoparasitic fungi can range from almost imperceptible (*Aegeritella*, *Hormiscium*, and *Laboulbenia camponoti*) to relatively weak in other Laboulbeniales [6] as manifested by size decline in the worker ants, behavioral changes, and even decreased lifespan of colony members [12,13]. The absence of strong negative effects in some associations likely reflects a long evolutionary history between these fungi and their ant hosts [1,6,14].

To date, 11 species of ectoparasitic fungi associated with ants have been reported from the Holarctic. However, Eastern Europe remains one of the most poorly studied regions in this regard, with only a small number of reports from Russia, Poland, Czech Republic, Bulgaria, Romania, Slovakia, Slovenia, Hungary and Croatia [6,14,15,16,17,18,19,20,21,22].

Holarctic representatives of ant-ectoparasitic fungi belong to the three phyla: Ascomycota, Basidiomycota, and Laboulbeniomycota. The species described here was found to be a member of the Ascomycota, class Dothideomycetes, order Capnodiales. Capnodiales originally included three families of sooty molds (Antennulariaceae, Capnodiaceae, Coccodiniaceae) [23]. Schoch et al. transferred Mycosphaerellaceae and Piedraiaceae into Capnodiales and recognized the Cladosporiaceae, thereby expanding the circumscription of this order [23]. Phylogenetic studies further enlarged the order, making it the second-largest order of Dothideomycetes. Based on this broad definition, Capnodiales was represented by 12 families: Capnodiaceae, Cladosporiaceae, Cystocoleaceae, Dissoconiaceae, Extremaceae, Mycosphaerellaceae, Neodevriesiaceae, Phaeothecaceae, Phaeothecoidiellaceae, Racodiaceae, Schizothyriaceae, Teratosphaeriaceae. However, recent studies using molecular genetic analysis have shown that Capnodiales s. lat. is polyphyletic and is represented by seven orders [24]. Three families are recognized in Capnodiales s. str., namely Capnodiaceae, Neoantennariellaceae and Readerielliopsidaceae.

According to polyphasic taxonomy, the family Readerielliopsidaceae includes the genera *Alloscorias* Haituk & Cheew, *Fumagospora* G. Arnaud, *Phaeoxyphiella* Bat. & Cif., *Readerielliopsis* Crous & Decock, *Scolecoxyphium* Cif. & Bat. and *Scorias* Fr. [24]. Representatives of this family are poorly studied in the context of insect associations, and their diversity and ecological roles remain insufficiently understood.

To date, only two species of ectoparasitic fungi associated with members of the ant genus *Lasius* are known: *Aegeritella tuberculata* Bałazy & J. Wiśn. and *Laboulbenia formicarum* Thaxt. The present paper reveals a third infectious agent for the genus *Lasius* belonging to a new species of a new genus of the family Readerielliopsidaceae. The myrmecoparasitic isolate was collected from the *Lasius distinguendus* worker ants in the Botanical Garden of the Southern Federal University (Rostov-on-Don, Russia). The new taxon was described using a multilocus molecular phylogenetic approach and morphological characteristics, and it is proposed here as a novel species belonging to a new genus. The pathogenesis pattern inside worker ants was studied using histological sections and micro-CT. The paper also discusses possible transmission pathways and the effect of the ectoparasite on the infected colony.

## 2. Materials and Methods

### 2.1. Collection and Culturing of Ants

The collection of *Lasius distinguendus* worker ants was carried out in the period from July 2021 to September 2024 from a single nest, which was located under a stone in *Thuja* plantings in the Southern Federal University Botanical Garden (SFEDU BG) (47°13′57.37″ N, 39°39′41.70″ E, Rostov-on-Don, Rostov Region of Russia). The live ants were collected and placed in standard test tube incubators. In total, 3 isolated incubators with around 10 worker ants in each were maintained for three months. The dead individuals were not removed from the test tubes during the entire experiment period. One incubator with worker ants of the same species collected in another location (Bagaevskaya village, Bagaevsky district, Rostov Region of Russia) was used as a control group. The ants were fed with 20% glucose syrup during the observation period.

### 2.2. Microscopy and Histological Studies

Light (Leica M205C, Leica DM4500 (Leica Microsystems CMS GmbH, Wetzlar, Germany)) and scanning electron (Hitachi TM3000 (Hitachi High-Technologies Corporation, Tokyo, Japan)) microscopes were used for morphological examination of infected ants (description and counting fungal bulbils on integuments of ants). For SEM, some individuals were preserved in 100% ethanol and then dried used the “critical point” method. The final images were processed with Adobe Photoshop CS5.

For histological studies live ants were fixed in alcoholic Carnoy’s solution and then embedded in paraffin, sectioned at 8–10 µm thickness using a microtome and stained with hematoxylin according to Boehmer’s method. To detect pathological changes in internal organs, uninfected individuals from the control group were subjected to the same procedures. Nine infected and three uninfected workers were examined.

### 2.3. Microtomographic (µCT) Studies

An array of microtomographic sections were obtained using a NeoScan N80 High Resolution Microtomograph (Neoscan, Mechelen, Belgium). Visualization, volume rendering, and segmentation of tomographic sections were performed in 3DSlicer 5.7.0 (https://www.slicer.org/). The ant body parts were scanned with the following parameters: voltage 38 kV, current 190 µA, without a filter, with a pixel size of 0.6 µm and a resolution of 4896 × 4896 pixels per slice with a continuous 360° rotation and a camera exposure of 145 ms per frame (3280 X-ray projections). The results of segmentation (in 3DSlicer) were saved in the file format STL. Next, Drishti v3.2.27.10.23 (National Computational Infrastructure, Australian National University, Canberra, Australia) software was used for visualization and animation of the ant body and fungal parts.

### 2.4. Strain Isolation

During field surveys of *L. distinguendus* ants in the SFEDU BG, visually distinguishable symptoms of infestation in the form of dark growths on the surfaces of the body were noted. It was suggested that these growths were caused by ectoparasitic fungi. Ants were examined under a stereoscopic microscope to screen for fungal growth. Ants with visible growths on the surface were washed three times with sterile saline and left to dry. Areas of the body and limbs with visually distinguishable growths were cut off with a sterile scalpel and then surface-sterilized with 96% ethanol for 1 min and flamed over an alcohol lamp.

These body parts of ants were placed on Petri dishes with agar media: potato carrot agar (PCA) [25], Sabouraud Agar (SDA) [26], Czapek agar (CZ) [27] and malt extract agar (MEA) [28]. Five pieces of ant parts were placed on each dish. In total, about 100 inoculations of ant parts were made. A control culture was prepared using uninfected ants from the same colony with the same cultivation method. All cultures were stored at room temperature in natural, diffused light. After 20–30 days of incubation, dark colonies were observed on all media with material from infected ants. In dishes with infected parts of ants, colonies were observed only near the body part or ant limbs. On negative control plates, dark colonies were not observed even after 60 days of incubation. The resulting cultures were transferred to sterile agar media for further cultural-morphological and molecular studies.

The isolated strain was deposited in the All-Russian Collection of Microorganisms (VKM), G.K. Skryabin Institute of Biochemistry and Physiology of Microorganisms at the Pushchino Biological Research Center of the Russian Academy of Science, Pushchino, Russia, under No. VKM F-5092. A dried type culture is preserved in the Mycological Herbarium of the Komarov Botanical Institute, St. Petersburg, Russia (acronym LE), under No. LE F-350982.

### 2.5. Molecular Studies and Phylogenetic Analysis

The pure culture was grown on CZ at 20 °C for 30 days for molecular analysis. DNA was extracted by using a Diamond DNA Plant kit (ABT, Russia, Barnaul) according to the manufacturer’s instructions. Internal transcribed spacer rDNA region (ITS1-5.8S-ITS2) was amplified using the PCR primers ITS1 (5′-TCCGTAGGTGAACCTTGCGG-3′) and ITS4 (5′-TCCTCCGCTTATTGATATGC-3′) [29]. The PCR amplification conditions were: 2 min at 95 °C, followed by 35 cycles, each consisting of 30 s at 95 °C, 30 s at 55 °C and 1 min at 72 °C; the final PCR extension step was 10 min at 72 °C. The D1/D2 region of 28S rDNA (LSU) was amplified using the PCR primers NL-1 (5′-GCATATCAATAAGCGGAGGAAAAG- 3′) and NL-4 (5′- GGTCCGTGTTTCAAGACGG -3′) [30]. The PCR amplification conditions were: 2 min at 94 °C, followed by 35 cycles, each consisting of 30 s at 94 °C, 1 min at 55 °C and 2 min at 72 °C; the final PCR extension step was 5 min at 72 °C. For amplification of the partial ribosomal polymerase II second-largest subunit (*RPB2*), primers 5F_Eur (5′-GAY-GAY-CGK-GAY-CAY-TTC-GG-3′) and 7CR_Eur (5′- CCC-ATR-GCY-TGY-TTR-CCC-AT-3′) were used [31]. The PCR cycling parameters were: 5 min at 94 °C, followed by 35 cycles, each consisting of 30 s at 94 °C, 30 s at 52 °C and 1 min at 72 °C; the final extension step was 10 min at 72 °C. The sequencing of the obtained DNA fragments was carried out using the Sanger method.

Sequences were trimmed, edited and assembled in BioEdit version 7.1.9. The obtained sequences were submitted to the NCBI GenBank, compared to available sequences in the database by using BLAST (https://blast.ncbi.nlm.nih.gov/Blast.cgi) (accessed on 16 August 2024). The sequences were aligned with other Capnodiales species sequences using the multiple sequence alignment ClustalW [32]. A combined LSU + ITS + *RPB2* data matrix containing 50 isolates belonging to 46 species was constructed. Data sets were created by combining the resulting sequences with preferably ex-type or holotype reference sequences from previous studies deposited at NCBI (Table 1). The phylogenetic analysis was performed by using the maximum likelihood method (ML) and Kimura 2-parameter model [33]. Initial tree(s) for the heuristic search were obtained automatically by applying Neighbor-Joining and BioNJ algorithms to a matrix of pairwise distances estimated using the Maximum Composite Likelihood (MCL) approach and then selecting the topology with a superior log likelihood value. The evolutionary history was inferred using the Maximum Parsimony (MP) method. The MP tree was obtained using the Subtree–Pruning–Regrafting (SPR) with search level 1 in which the initial trees were obtained by the random addition of sequences (10 replicates) [34]. The partial deletion option was applied to eliminate all positions with less than 95% site coverage, resulting in a final data set. Confidence levels in nodes were determined using bootstrap analyses of 1000 replicates.

### 2.6. Morphological Analysis

For morphological analysis the isolates were cultivated on Czapek agar (CZ) [27], malt extract agar (MEA) [28], Sabouraud agar (SDA) [35] and potato carrot agar (PCA) [25]. The isolates were inoculated on 9 cm Petri dishes and incubated for 30 days at 21 °C in darkness. In addition, inoculated CZ, MEA and SDA plates were incubated for 21 days at 2, 6, 8, 12, 16, 20, 25, 30, 32 and 35 °C. Color determination was performed according to the ISCC-NBS Centroid Color Charts [36], according to the recommendations of Nováková et al. [37]. Microscopy by Carl Zeiss Axio Imager A1 was used for micromorphological examination.

### 2.7. Enzymatic Activity

Tests for enzymatic activity (hemolytic, phospholipase, chitinase, ligninolytic and cellulolytic activity) were carried out according to the methods described in our works [38,39].

## 3. Results

As a result of the research, one strain belonging to one previously undescribed species and the genus *Xenomorphomyces* **gen. nov** was isolated.

A BLAST analysis of LSU sequences demonstrated a relationship between the species *X. formicartus* and *Readerielliopsis guyanensis* (Decock) Crous & Decock, with a 95.20% similarity of the sequences. According to a BLAST analysis of the ITS region, *X. formicartus* is most closely related to *R. guyanensis* with an 89.35% similarity. A BLAST analysis of the partial *RPB2* gene demonstrated a relationship between the species *X. formicartus* and *Scorias spongiosa* (Schwein.) Fr., with an 80.56% similarity to the sequences.

**Figure 1:**
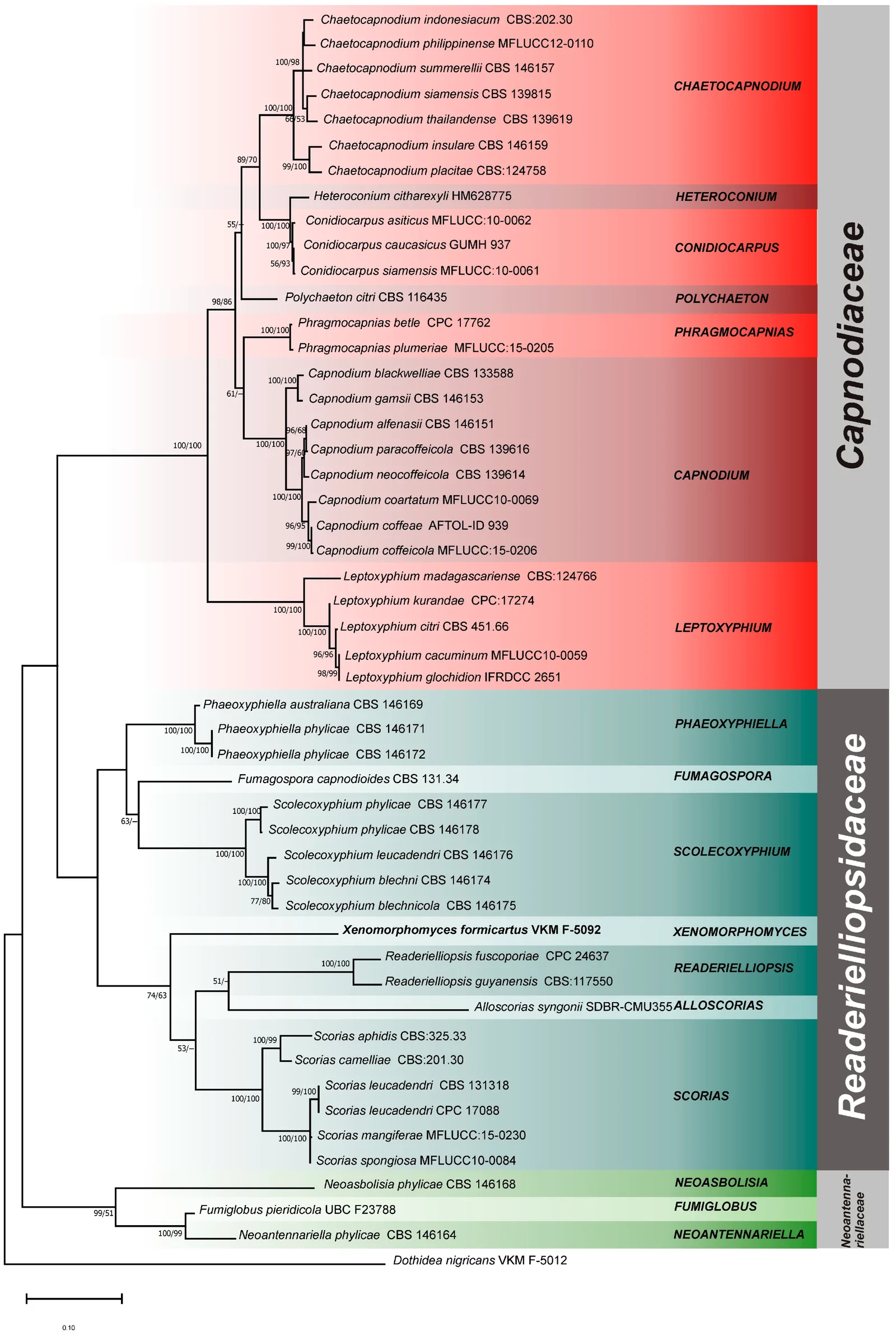
Phylogenetic tree of the combined ITS + LSU + *RPB2* sequences showing the relationships between *Xenomorphomyces formicartus* and the other species in Capnodiales. Bootstrap support values (1000 replicates) for maximum likelihood (ML) and Maximum Parsimony (MP) are shown at the nodes as ML/MP. The tree is rooted with *Dothidea nigricans* VKM F-5012. Values below 50% are not shown (or replaced with —).

Sequence data from LSU, ITS and *RPB2* were analyzed for examination of the *X. formicartus* phylogeny. The data of 50 isolates including the outgroup (*Dothidea nigricans* VKM F-5012) were used to reveal the phylogenetic relationships of the supposed new species with other species of Capnodiales. The following loci were included in the phylogenetic analysis: the partial 28S rDNA gene (LSU) (571 sites/145 variable), the ITS region (449 sites/238 variable), and the partial *RPB2* gene (579 sites/289 variable). There were a total of 1599 positions in the final data set. The combined maximum likelihood phylogenetic tree based on LSU, ITS and *RPB2* is shown in Figure 1.

According to this analysis, *X. formicartus* clustered together with *Readerielliopsis guyanensis*, *R. fuscoporiae* Crous & Decock, *Alloscorias syngonii* Haituk & Cheew *and Scorias* spp. as the most closely related species.

****

**Taxonomy**

****

***Xenomorphomyces*** V.A. Iliushin & I.Y. Kirtsideli, **gen. nov.**

MB 856516

**Etymology.** Referring to the parasitic organism “Xenomorph” from the science fiction movie “Alien” + the ending “-myces” is a standard suffix meaning “fungi”.

**Type species. ***Xenomorphomyces formicartus* V.A. Iliushin & I.Y. Kirtsideli

**Figure 2:**
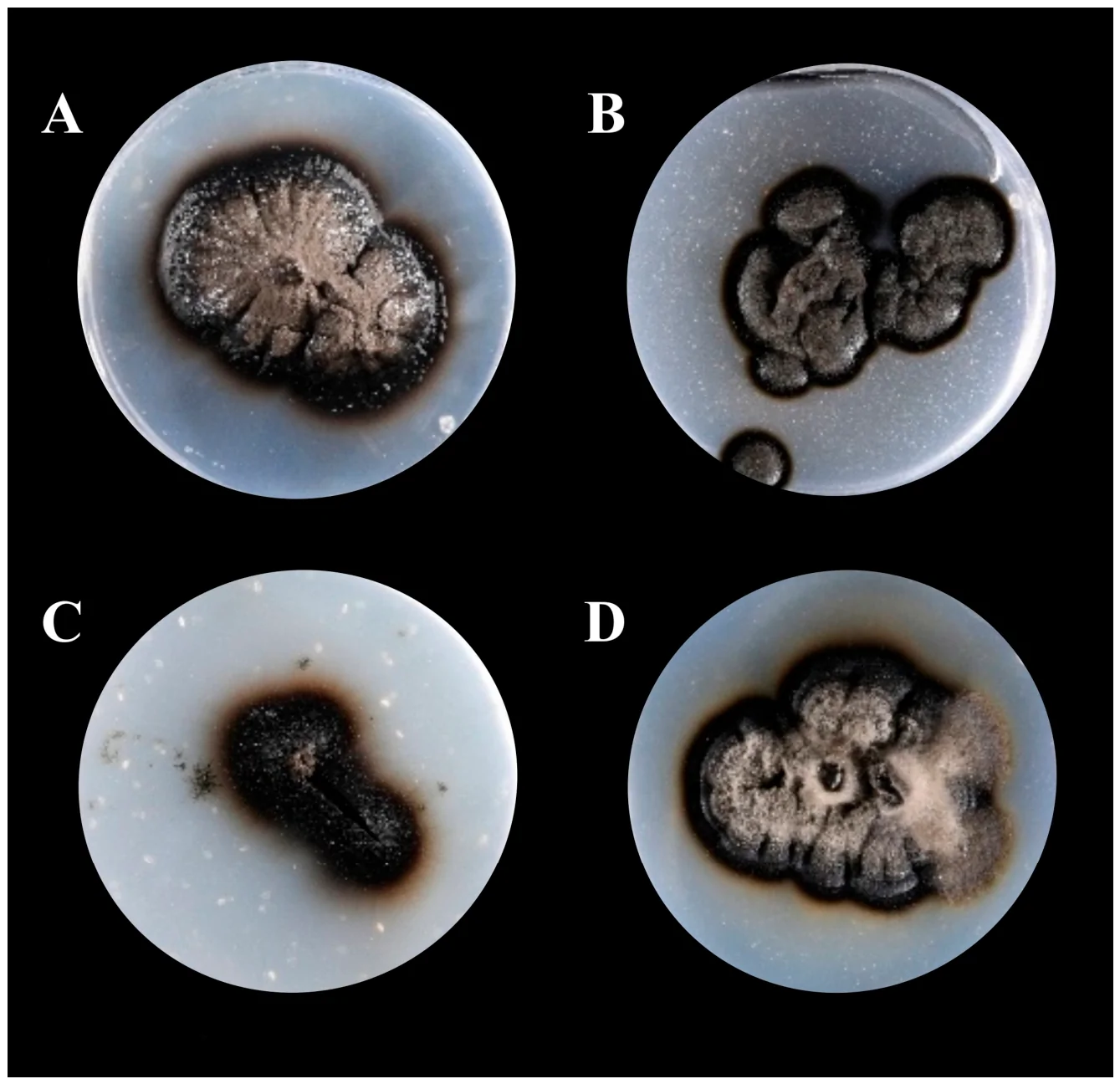
Macromorphology of *Xenomorphomyces formicartus*. Morphogenesis of colony growing on SDA (**A**), CZ (**B**), PCA (**C**) and MEA (**D**) at 20 °C. Petri dishes, 60 mm.

**Figure 3:**
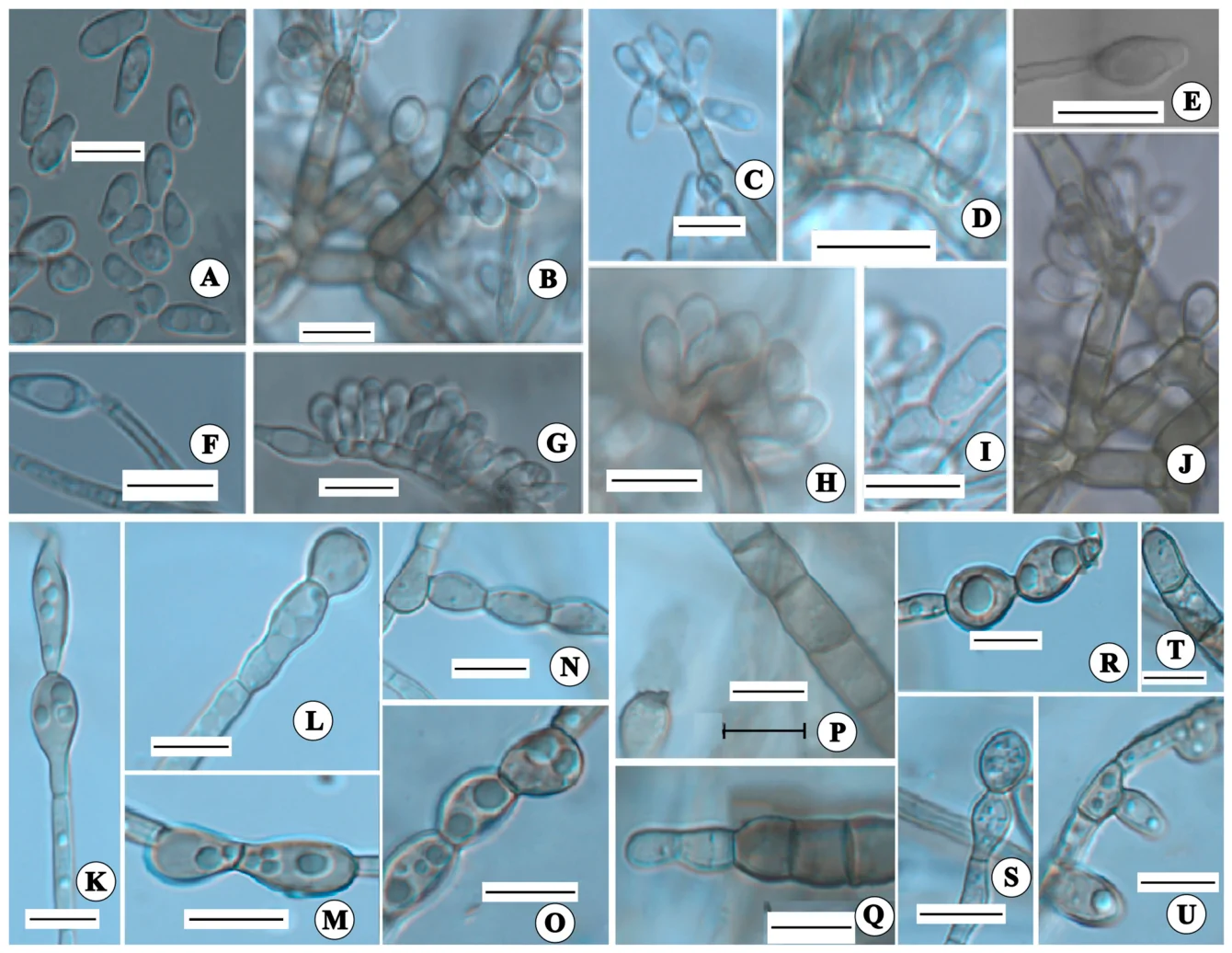
Micromorphology of *Xenomorphomyces formicartus*, (**A**–**J**)—conidia and conidiophores; (**K**–**U**)—mycelium and chlamydospores. Scale bars—10 μm.

***Xenomorphomyces formicartus*** V.A. Iliushin & I.Y. Kirtsideli, **sp. nov.** (Figure 2 and Figure 3)

MB 856517

***Generic-specific diagnosis.*** The mycelium is hyaline to light brown and septate; the walls are smooth, sometimes slightly rough, branching. It forms multiple chlamydospores, both at the ends of hyphae and intercalary ones, often in chains ranging in size. Tangles of hyphae are rare.

**Type:** RUSSIA. Rostov Region: Rostov-on-Don, Botanical Garden of the Southern Federal University, (47°24′7.289″ N, 39°39′59.599″ E): from ants, isol. I.Y. Kirtsideli, (holotype LE F-350982 dried culture); ex-type culture VKM F-5092. ITS barcode: PQ555703 (alternative markers: LSU = PQ555705; *RPB2* = PQ565711).

**Etymology:** The name refers to the association with ants, “formi” for an ant and “cartus” for a container.

**Colony characters:** The appearance of dark colonies in dishes with infected parts of ants was observed only near the body part and limbs of the ant. Colonies were found on 14% of the analyzed samples. A total of eight fungal cultures were isolated as pure cultures, which did not differ in morphological characteristics. No significant differences were noted in the infected parts of the ants’ bodies from which the colonies were isolated.

*Macromorphology.* Colonies on Sabouraud medium (Figure 2A) reach 46 mm after 30 days at 20 °C. The colony is zoned; the edge is black and slightly lighter towards the center, with a velvety coating. The substrate mycelium is dark brown; the aerial mycelium is brown and colorless. The reverse is dark brown; the medium is not colored. Exudate is absent.

Colonies on Czapek medium reach 38 mm after 30 days at 20 °C. The colony is zoned; the edge is wide and dark brown, light brown towards the center, and felt-like. The substrate mycelium is brown; the aerial mycelium is mainly gray-brown. The reverse is dark brown or black; the medium is not colored. Exudate is absent. Some satellite colonies formed near the edge of the mother colony (Figure 2B).

On PCA (Figure 2C) they reach 34 mm after 30 days at 20 °C. The colony is not zoned and is semi-submerged in the substrate. Exudate is absent. The substrate mycelium is dark brown; the aerial mycelium is brown. The reverse is dark brown; the medium is not colored.

On MEA (Figure 2D) they reach 45 mm after 30 days at 20 °C. The colony is zoned; the edge is dark brown, wide, light brown towards the center, and felt-like. Exudate is absent. The substrate mycelium is dark brown; the aerial mycelium is colorless. The reverse is dark brown or black; the medium is not colored.

Growing at different temperatures does not affect the color of the colonies.

*Micromorphology.* Microscopic examination showed that most of the fungal colonies consist of septate mycelium and a large number of chlamydospores. Conidial sporulation begins to form only after 20–30 days of growth, usually in the upper part of the colony in the aerial mycelium zone.

Conidial sporulation is hyaline, from round-oval to oval-elongated, often pear-shaped, 2.2–5.3 × 8.2–12.5 (13.5) µm. Conidia are unicellular (Figure 3A) and less often two- or three-celled (Figure 3I). Conidiophores are single (Figure 3E,F) and can form a cluster of conidia at the end of the conidiophores (Figure 3C). Branching conidiophores are noted (Figure 3B,J), where the formation of conidia occurs both at the end of the conidiophores and at the sites of branching of the conidiophores. The formation of structures resembling a bed (a cluster of conidiophores growing next to each other) can also be noted (Figure 3D,G,H). Pycnidia are absent.

The mycelium is hyaline to light brown and septate; the walls are smooth, sometimes slightly rough, branching, from 2.5 to 7.2 µm (Figure 3K,P,S,T). It forms multiple chlamydospores (both at the ends of hyphae and intercalary), often in chains, ranging in size from 5.3 to 14.1 µm, often darkly pigmented (Figure 3L–O,Q,R). Tangles of hyphae are rare.

These characteristics of this fungus suggest that this fungus is likely capable of disseminating such dispersed cells as propagules, in addition to forming conidia.

Note. The genus *Xenomorphomyces* was described based on fungal isolates recovered from an ant colony. This genus differs significantly ecologically and morphologically from other genera in the family Readerielliopsidaceae. Most members of these fungi are epiphytic and belong to the so-called sooty molds, which form a dark coating on the surfaces of living plants. They feed primarily on the sugary secretions of aphids and other insects, not directly parasitizing the tissues of living plants but impairing their photosynthesis by forming a dense black coating. The genus *Xenomorphomyces* (*X. formicartus*) is described as a parasite of *Lasius distinguendus*, i.e., it can be considered a fungal infection of the ant and an entomopathogen of insects. From an ecological and evolutionary point of view, this immediately distinguishes the genus *Xenomorphomyces* from other representatives of the family Readerielliopsidaceae.

The main differences between the genus *Xenomorphomyces* and all other genera in the family Readerielliopsidaceae concern the absence of pycnidia and the branching of conidiophores. In all other genera (*Alloscorias*, *Scolecoxyphium*, *Fumagospora*, *Scorias*, *Phaeoxyphiella*), pycnidia are invariably present and vary in shape—from spherical and pear-shaped to elongate-cylindrical, horn-like, or flask-shaped. By contrast, *Xenomorphomyces* completely lacks pycnidia; instead, it forms characteristic “bed-like” structures—dense clusters of conidiophores growing side by side.

The second major distinction is the conidiophore morphology. In most genera, conidiophores are reduced to single conidiogenous cells, with only *Fumagospora* and *Phaeoxyphiella* showing weakly branched forms. *Xenomorphomyces*, however, has distinctly branching conidiophores, which further sets it apart within the family. In terms of conidia, the genus does not differ from the rest: conidia range from hyaline to brown, are round-oval to oblong, ellipsoid or elongated, and may be single-celled or possess transverse and longitudinal septa (as seen in *Fumagospora* and *Phaeoxyphiella*). The main differences between the genera of the family are presented in Table 2. The identification key to the genera of Readerielliopsidaceae is presented in a separate section below.

**Figure 4:**
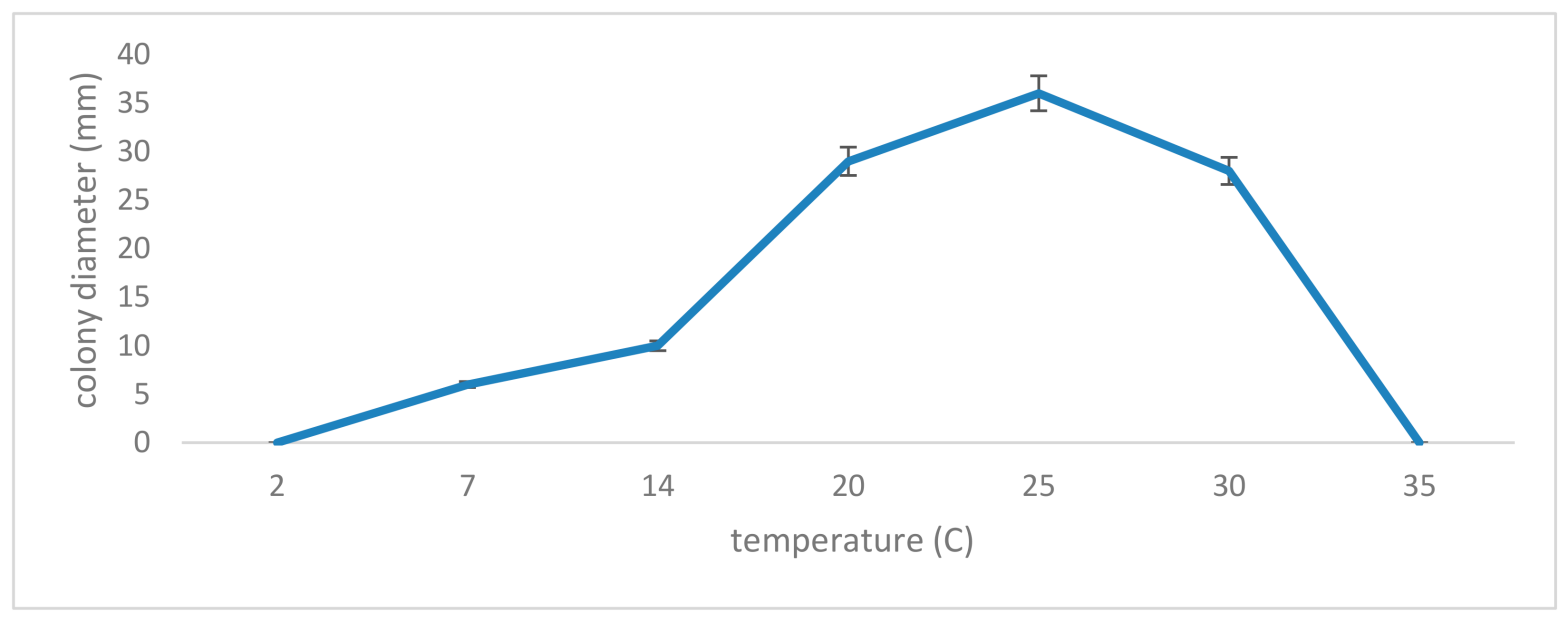
Average colony diameter of *Xenomorphomyces formicartus* assessed on SDA after 25 d growth in the dark at temperatures ranging from 2 to 35 °C.

*Physiology*. Cultural properties of strains of *X. formicartus* grown on different media were examined. The growth test on various media of neutral pH showed that *X. formicartus* was able to grow on all the media tested, and the best hyphal growth was on SDA and MEA plates (Figure 2A,D). Growth reduction was clearly shown when grown on PCA plates (Figure 2C). Vegetative growth and conidial reproduction at various conditions of temperature were examined in our strains of *X. formicartus*. The growth tests at different temperatures showed that all the strains were able to grow in the range of 7–30 °C, with better growth at 25 °C. No visible hyphal growth was detected at either 2 °C or 35 °C (Figure 4). Examination at various temperatures suggests that *X. formicartus* is a mesophilic organism.

The isolate *X. formicartus* was tested for extracellular enzymatic activities. Hemolytic, phospholipase and chitinase activity was detected; ligninolytic and cellulolytic activity was not noted.

*Pathology*. To date, the observed colony of *L. distinguendus* in SFEDU BG is the only one found to be infected with *X. formicartus*. A survey of nearby areas and excavation of ant nests did not reveal any additional infected colonies. Even the ants from the colony of *Lasius neglectus* Van Loon, Boomsma & Andrasfalvy, 1990 found under a nearby stone showed no signs of infection with the fungus (more than fifty workers and several queens were examined under a stereomicroscope).

**Figure 5:**
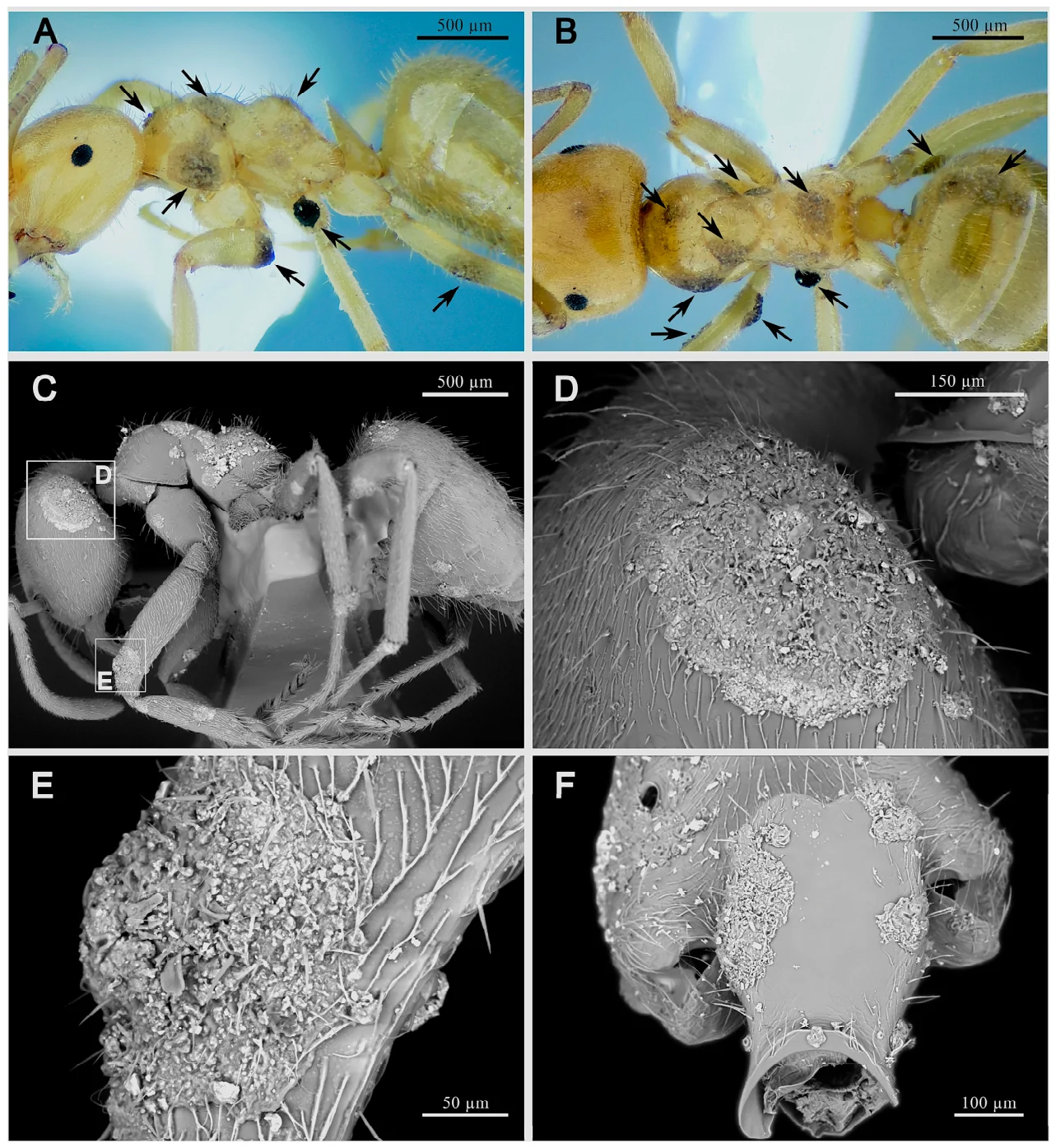
Fungal bulbils on the body of *L. distinguendus* ant. (**A**,**B**)—photomicrographs; (**C**–**F**)—SEM micrographs.

Over a four-year observation period, the proportion of infected individuals within the colony remained consistently close to 100% (groups of about fifty workers were selected several times a year). Fungal colonies on the cuticle were visible as brown-black oval bulbils of 0.1–0.6 mm in diameter and 0.03–0.2 mm thick (Figure 5). The fungal colonies grow chaotically on all parts of the ants’ bodies except the antennae and the distal sections of the legs. The most frequent growth sites were observed on the gaster and thorax. Sometimes the fungus covered almost the entire cuticle area, developing especially strongly on the abdomen. Young fungal colonies (we mean those that were visually smaller in diameter) appear lighter in color, whereas mature, larger colonies are darker and melanized (Figure 5A,B). In spring, it was observed that a higher proportion of ants carried younger (lighter and smaller) fungal colonies compared to the summer and autumn months. The percentage of uninfected (without visual signs of infection) individuals was also higher in spring.

The maximum lifespan of the infected workers under laboratory condition was about 1–2 months, while the lifespan of the uninfected *L. distinguendus* workers was more than 8 months. The dead infected individuals were placed into the incubator with the uninfected ants, but they immediately took them to the marginal dry part of the incubator and did not come close to them for a long time. No infected workers were found within three months after the experiment.

**Figure 6:**
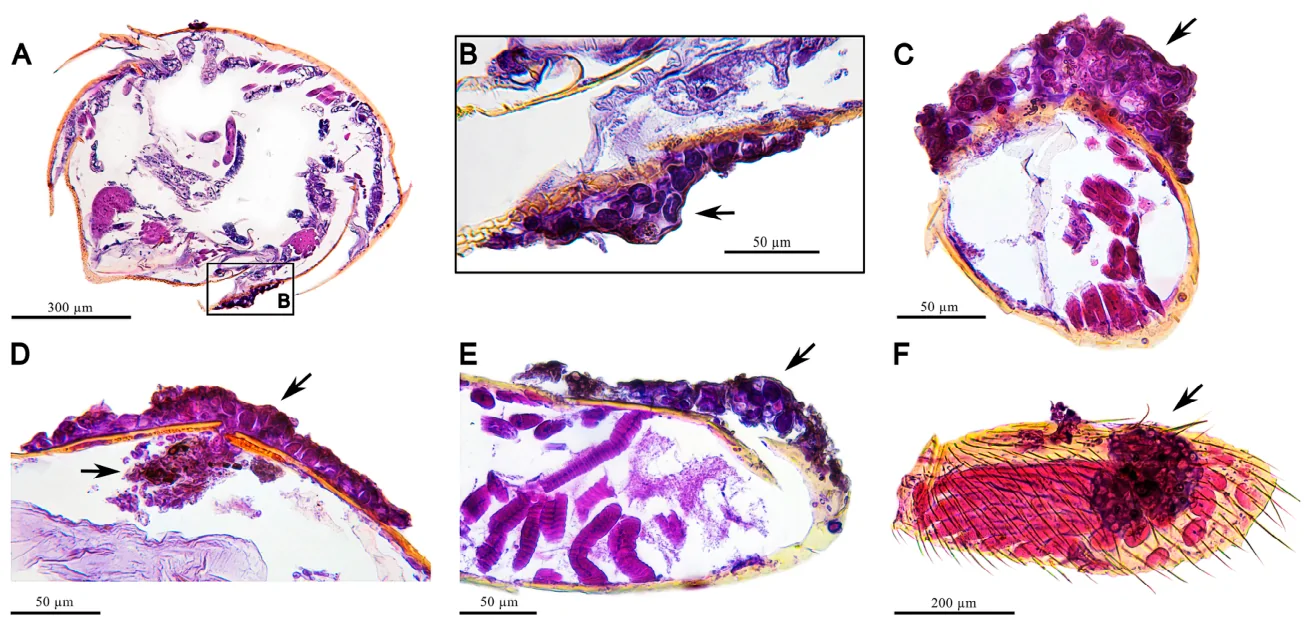
Histological examination of fungal bulbils on the body parts of ant *L. distinguendus*. (**A**,**B**,**D**)—on the abdomen; (**C**,**E**,**F**)—on the legs.

Histological studies showed that the fungal colonies grow mostly on the surface of the cuticle. In two slides the penetration of the mycelium of the fungi under the cuticle of the gaster was observed: a small round hole under the center of the colony allowed *X. formicartus* to enter the body cavity (Figure 6C,D).

**Figure 7:**
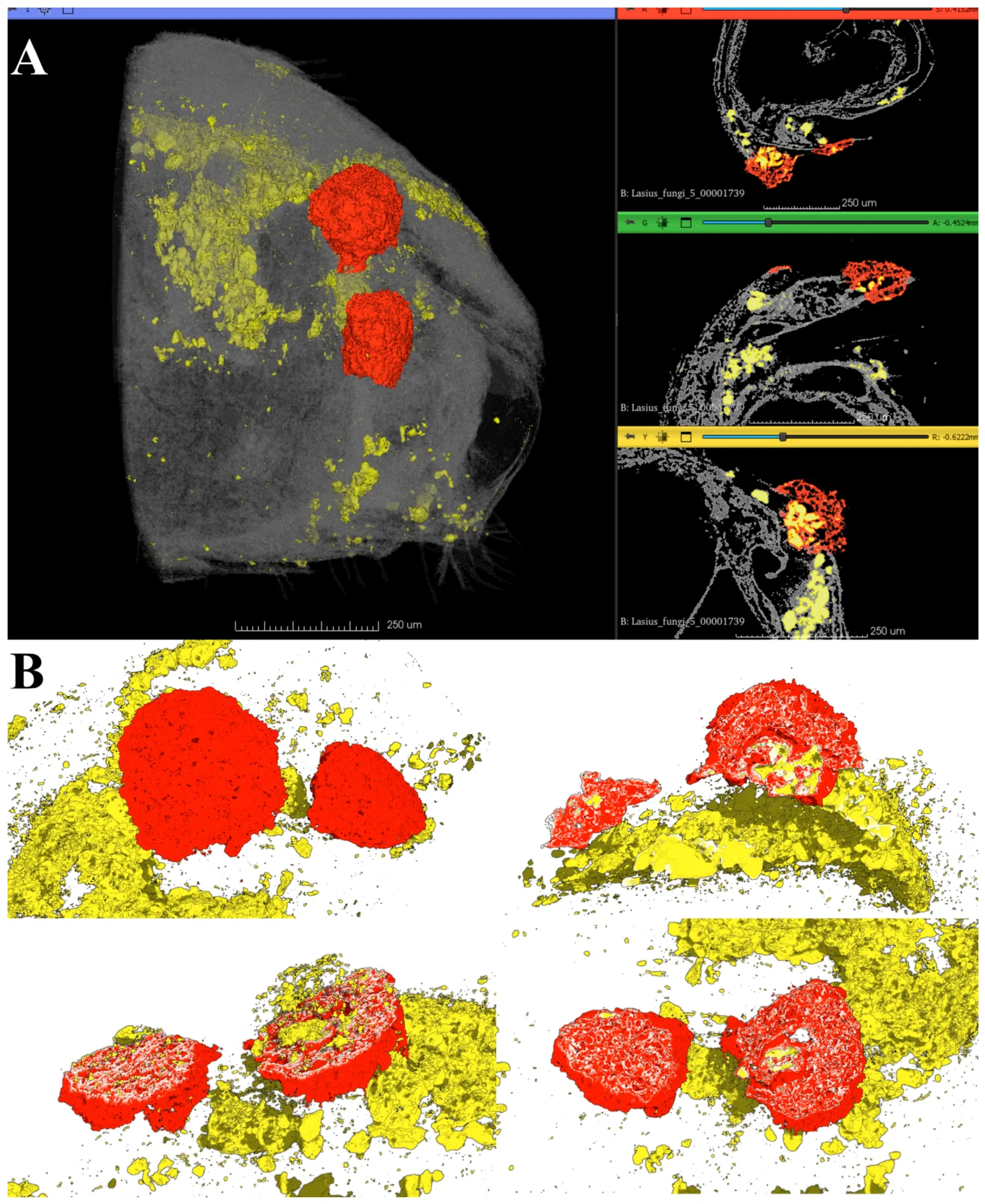
µCT volume rendering (**A**) and 3D modeling (**B**) of the fungal structures on the abdomen of *L. distinguendus*. Red—fungal bulbils (on the surface of abdominal sclerites), yellow—fungal mycelium. Also see Supplementary Material (Video S1, video file).

The results of X-ray microtomographic examination of the body parts affected by the fungus (head, metasoma and gaster) show that the fungal mycelium is located directly under the integuments of the ants (Figure 7, marked in yellow). The “living” mycelium (when samples are dried using the critical point method) of the fungus and bulbils on the surface of the sclerites have different X-ray densities and are clearly distinguishable during rendering (see Supplementary Material Video S1). Part of the mycelium is located inside the bulbils. The microtomographic sections from volumetric rendering (Figure 7A, Supplementary Material Video S1) and the resulting 3D models (Figure 7B, Supplementary Material Video S1) clearly show the spongy structure of bulbils with mycelium embedded in them.

**Identification key to the genera of Readerielliopsidaceae**
** **
This key is a supplemented and modified version of the identification key to the genera of the family Readerielliopsidaceae by Haituk et al. [40].
** **

**1.** Growing on ants, producing dense bulbils on the insect’s body ……………………………………………………………………………***Xenomorphomyces***– Growing on leaves, forming superficial hyphal networks or mycelial masses ………………………………………………………………………………….2**2.** Forming a delicate superficial weft of compact, dark-colored hyphae on leaves ………………………………………………………………………………3– Forming a black, arachnoid (web-like) mycelial aggregation on leaves ………………………………………………………………………………………….6**3.** Pycnidia ampulliform (flask-shaped), subspherical to pyriform, brown ………………………………………………………………………………………..4– Pycnidia irregularly cylindric, straight or sinuous, short to elongate ………………………………………………………………………….***Scolecoxyphium*****4.** Conidia globose to clavate, aseptate (non-septate) ………………………………………………………………………….…………………..***Readerielliopsis***– Conidia septate ………………………………………………………………………………………………………………………………………………………….5**5.** Conidia thread-like (filiform), fusiform-ellipsoid, with up to 15 septa ………………………………………………………………………..***Phaeoxyphiella***– Conidia oblong or ovoid, with 3–4 transverse septa …………………………………………………………………………….…………………..***Fumagospora*****6.** Ascostromata stalked; ascospores oblong to saccate, 3-septate, hyaline …………………………………………………………………………………***Scorias***– Ascostromata sessile (without stalks); ascospores fusoid, 3-septate, enveloped by a mucilaginous sheath, hyaline ………………………….***Alloscorias***


## 4. Discussion

The family Readerielliopsidaceae was separated into seven genera. A combined tree based on ITS + LSU + *RPB2* partial sequences showed that *X. formicartus* is also included in Readerielliopsidaceae and is most closely related to *Readerielliopsis guyanensis*, *R. fuscoporiae*, *Alloscorias syngonii* and *Scorias* spp. *Readerielliopsis fuscoporiae* was isolated from basidiomata of *Fuscoporia wahlbergii*; *R. guyanensis* was isolated from a decaying leaf of an angiosperm [41,42]. The finds were made in South America (French Guiana). *Alloscorias syngonii* was isolated from living leaves of the plant *Syngonium podophyllum* in Thailand [40]. Species of the genus *Scorias* have been isolated from living leaves of diverse plant hosts (*Coffea*, *Citrus*, *Gardenia*, *Ficus*, *Fagus*, and others) in Brazil, Taiwan, the Philippines, South Africa, and North America [43].

*X. formicartus* has unique sequences of ITS, LSU and *RPB2*. The low degree of genetic relatedness and the formation of a distinct subclade indicate that the fungus should be classified as a separate genus.

This finding adds to the growing body of evidence that associations between fungi and ants have evolved independently multiple times across different lineages. Recently, Siedlecki et al. reached a similar conclusion with the discovery of *Formicomyces microglobosus* in Trichomeriaceae, which strengthened the hypothesis of multiple, independent evolution of ant-associated fungi [44]. Our phylogenetic data support this broader evolutionary pattern, suggesting that ant-associated lifestyles have arisen convergently in distinct fungal families.

Morphological characteristics also do not allow us to classify it among previously described species and genera of fungi. *Xenomorphomyces* is set apart from the rest of the Readerielliopsidaceae by the absence of pycnidia and by conidiophore branching.

The Capnodiales include fungi known as “sooty molds” with diverse lifestyles and nutrition habits. These fungi include saprobes, plant and animal pathogens, mycoparasites, rock-inhabiting fungi, lichenized fungi, and endophytes. The vast majority of the species of the Readerielliopsidaceae are inhabitants of plants. However, the majority of these fungi are not phytoparasitic. Their development is directly dependent on the excretions of sap-feeding insects, particularly aphids [24]. Fungi belonging to the genus *Scorias* utilize sugar-rich honeydew as their primary nutritional substrate. For instance, *Scorias spongiosa* in North America grows exclusively on the honeydew produced by the aphid *Grylloprociphilus imbricator*, which infests the American beech (*Fagus grandifolia*). Consequently, the distribution of these fungi is closely correlated not only with their host plants but also with their associated insect symbionts [43].

In the exoenzyme assays, *X. formicartus* exhibited hemolytic, phospholipase, and chitinase activities, whereas ligninolytic and cellulolytic activities were not detected. Although the presence of chitinase may suggest a potential for cuticle interaction, such enzymatic activity alone does not demonstrate entomopathogenicity. Therefore, the enzyme profile is more cautiously interpreted as indicative of an insect-associated lifestyle rather than of saprotrophic involvement in soil organic matter decomposition. However, the fungus should not be conclusively classified as an entomopathogen based solely on these biochemical data. Its ability to cause disease in ants, as well as its transmission pathways, remains to be confirmed by controlled infection experiments. Consequently, the observed fungal growth on *L. distinguendus* workers is best described as an association, and the possible pathogenic effects and transmission routes discussed above should be regarded as working hypotheses requiring further investigation.

Although the infection rate in the observed *L. distinguendus* colony is consistently close to 100%, the colony has successfully thrived for over five years (the last observations of the colony were conducted in April 2026). This indicates that *X. formicartus* probably has a moderate negative impact on the ants, suggesting low lethality. The absence of strong negative effects likely reflects a long evolutionary history of interaction between these fungi and their hosts. However, laboratory observations suggest that the fungus may shorten the ants’ lifespan, even if its overall impact in natural conditions seems limited. It also remains unclear whether the queen in the colony is infected and how the fungus affects its reproductive functions and life expectancy.

The fungus predominantly develops on the surface of the ant’s cuticle without invading the host’s body cavity or damaging internal organs.

In the later stages of development, the fungus can pierce the cuticle, penetrating the body cavity of the host. This can occur due to turgor pressure or with the help of secreted enzymes (this is indirectly confirmed by chitinase activity detected in laboratory cultures of *X. formicartus*), which aligns with observations of other entomopathogenic fungi, where the production of hydrolytic enzymes, including chitinases, is a key factor in cuticle penetration [45]. According to our observations, the stage at which the fungus invades the body cavity occurs approximately 3–4 months after infection and presumably causes temperate and potentially severe effects. Still, the period is long enough for new generations of worker ants to emerge and for the colony to grow. Nevertheless, the fungus causes stiffening of the limb joints, head, and mouthparts, as it often grows in these areas of the cuticle (Figure 5). This can severely impair the ants’ ability to move and feed.

Within an ant colony, infection is likely transmitted through direct contact between individuals. Since the observed colony is the only recorded case of parasitism by *X. formicartus*, we can only hypothesize that transmission between colonies may occur during nuptial flights via alates. The fact that close relatives (*Lasius* s. str.), whose colonies were in direct contact with the infected one, were not infected by the fungus may be due to high host specificity or to difficulties in colony-to-colony transmission. Infection of uninfected individuals of the same species could not be reproduced under experimental conditions, possibly because the ants discard infected cadavers and avoid close contact with them. It was also not possible to house live infected ants with uninfected ones, since individuals from different colonies exhibited high aggression towards each other, which in most cases resulted in the death of the infected individual; afterwards, the cadaver was also removed to the periphery of the incubator. It is possible that environmental conditions, especially humidity, may also influence the spread of infection under both laboratory and natural conditions.

When observed on the surface of the ant host, the lesions caused by *X. formicartus* and *Aegeritella* species show considerable morphological overlap, which can complicate visual identification during surveys. However, these two genera are well distinguishable with proper mycological examination; a detailed analysis of spore morphology and molecular markers provides clear diagnostic features. It is therefore a possibility that some reports of *Aegeritella* in Europe could be misidentified as instances of *Xenomorphomyces*. Therefore, any survey of ant-associated fungi should incorporate comprehensive mycological data to avoid misidentification, given that the external lesions are superficially similar [1].

*Xenomorphomyces formicartus* was isolated from multiple individuals within a single ant colony, with a relatively high prevalence of infection. Although this sampling limitation does not undermine the taxonomic validity of the new species and genus—which is firmly supported by morphological characters and multilocus phylogenetic analyses—it prevents us from making definitive statements about the fungus’s distribution, ecology, or pathogenesis. Accordingly, our observations on these aspects should be regarded as preliminary and interpreted with caution.

Since botanical gardens around the world are sources of biological invasions, it remains uncertain whether the presence of *X. formicartus* in the SFEDU BG represents a biological invasion or whether the species is native to the region. If some reports of *Aegeritella* from Eastern Europe turn out to be *Xenomorphomyces*, this would support the hypothesis that the fungus is native to southern Russia and adjacent regions. Nevertheless, further genetic and ecological studies are required to clarify the species’ origin and potential dispersal pathways.

## 5. Conclusions

The present work extends our knowledge of mycosis in ants, provides data on a new parasitic fungal species and genus, and evaluates its phylogenetic position showing its close relationship with representatives of Readerielliopsidaceae. Although the initial information on the spread of the parasite was obtained, the method of transmission of *Xenomorphomyces* requires further investigation. Our data indicate that the fungus can be clearly distinguished from other species based on phylogenetic and morphologic analyses and represents the novel species *Xenomorphomyces formicartus* in the genus *Xenomorphomyces*.

## Figures and Tables

**Table 1:** Strains included in the phylogenetic analysis and their GenBank accession numbers. The *X. formicartus* sequences are in bold. “nd”—no data.

Species	Collection No.	LSU	ITS	*RPB2*
*Alloscorias syngonii*	SDBR-CMU355	MW263929	MW263927	MW269550
*Capnodium alfenasii*	CBS 146151	MN749165	MN749233	MN829260
*C. blackwelliae*	CBS 133588	MH878118	MN749235	GU371743
*C. coartatum*	MFLUCC10-0069	JN832614	nd	nd
*C. coffeae*	CBS 147.52	GU214400	DQ491515	KT216519
*C. coffeicola*	MFLUCC15-0206	KU358920	KU358921	nd
*C. gamsii*	CBS 892.73	GU301847	MN749237	GU371736
*C. neocoffeicola*	CBS 139614	MN749172	MN749242	MN829267
*C. paracoffeicola*	CBS 139616	MN749174	MN749244	MN829269
*Chaetocapnodium* *indonesiacum*	CBS 202.30	GU301849	MH855113	MN829273
*C. insulare*	CBS 146159	MN749178	MN749248	MN829274
*C. philippinense*	MFLUCC12-0110	KP744503	MN749251	MN829277
*C. placitae*	CBS 124758	GQ303299	GQ303268	MN829278
*C. siamensis*	CBS 139815	MN749181	MN749252	MN829279
*C. summerellii*	CBS 146157	MN749176	MN749246	MN829271
*C. thailandense*	CBS 139619	MN749183	MN749254	MN829281
*Conidiocarpus asiticus*	MFLUCC10-0062	JN832612	KU358924	nd
*C. caucasicus*	GUMH 937	KC833050	nd	nd
*C. siamensis*	MFLUCC10-0061	JN832607	KU358923	nd
*Fumagospora capnodioides*	CBS 131.34	EU019269	AJ244240	KT216553
*Heteroconium citharexyli*	HM628775	HM628775	HM628776	nd
*Leptoxyphium cacuminum*	MFLUCC10-0059	JN832603	nd	nd
*L. citri*	CBS 451.66	KF902094	MN749266	GU371727
*L. glochidion*	IFRDCC 2651	KF982308	KF982307	nd
*L. kurandae*	CBS 129530	JF951170	JF951150	MN829295
*L. madagascariense*	CBS 124766	MH874923	MH863407	MN829296
*Neoantennariella phylicae*	CBS 146164	MN749209	MN749281	MN829311
*Neoasbolisia phylicae*	CBS 146168	MN749215	MN749287	MN829317
*Phaeoxyphiella australiana*	CBS 146169	MN749220	MN749292	MN829322
*P. phylicae*	CBS 146171	MN749216	MN749288	MN829318
*P. phylicae*	CBS 146172	MN749217	MN749289	MN829319
*Readerielliopsis fuscoporiae*	CBS 139900	KR476755	KR476720	MN829326
*R. guyanensis*	CBS 117550	FJ493211	MH863023	MN829327
*Scolecoxyphium blechni*	CBS 146174	MN749224	MN749296	MN829328
*S. blechnicola*	CBS 146175	MN749225	MN749297	MN829329
*S. leucadendri*	CBS 146176	MN749226	MN749298	MN829330
*S. phylicae*	CBS 146177	MN749227	MN749299	MN829331
*S. phylicae*	CBS 146178	MN749228	MN749300	MN829332
*Scorias aphidis*	CBS 325.33	MH866910	GU214696	KT216542
*S. camelliae*	CBS 201.30	MH866560	MH855112	MN829333
*S. leucadendri*	CBS 131318	JQ044456	JQ044437	MN829334
*S. mangiferae*	MFLUCC15-0230	KT588603	KT588604	nd
*S. spongiosa*	MFLUCC10-0084	JN832601	nd	nd
*Xenomorphomyces formicartus*	**VKM F-5092**	**PQ555705**	**PQ555703**	**PQ565711**
Outgroup: *Dothidea nigricans*	VKM F-5012	OR801565	OP961959	OR810751

**Table 2:** The main differences between the genera of the family *Readerielliopsidaceae*.

Genera of the Family *Readerielliopsidaceae*	Habitats	Conidia	Conidiophores	Pycnidia
*Alloscorias*	Form hyphal masses, black, web-like colonies on leaves	Conidia are unicellular, oblong to ellipsoid or teardrop-shaped, hyaline, and smooth-walled	Conidiophores are reduced to conidiogenous cells	Pycnidia are spherical to subspherical
*Fumagospora*	Developing on the leaf surface, a network of dense, dark hyphae	Conidia are partially swollen, brown, of varying shapes, oblong or ellipsoid, with transverse and longitudinal septa	Conidiophores are very short, simple (unbranched) or weakly branched at the base, colorless (hyaline), cylindrical or filiform	Pycnidia are elongated, cylindrical or subcylindrical, resembling long stalked structures or “horns”
*Phaeoxyphiella*	Developing on the leaf surface, a network of dense, dark hyphae	Conidia with septa (up to 15) are large and strongly elongated	Conidiophores are hyaline, smooth, cylindrical or subcylindrical, separated by septa or simple	Pycnidia are dark brown or black, spherical, slightly flattened, pear-shaped, or conical
*Readerielliopsis*	Developing on the leaf surface, forming a network of dense, dark hyphae. Isolated from the fruiting bodies of tinder fungi	Conidia are single-celled, brown, smooth, and spherical to club-shaped	Conidiophores are reduced to conidiogenous cells	Pycnidia are brown, subspherical to pear-shaped
*Scolecoxyphium*	Developing on the leaf surface, a network of dense, dark hyphae	Conidia are hyaline, small, mostly elongated, oval, elliptical, or bacilliform (rod-shaped). Typically without septa	Conidiophores short, hyaline, cylindrical or flask-shaped	Pycnidia are irregularly cylindrical, straight or sinuous, short or long, without a sharp widening in the spore formation zone
*Scorias*	Form hyphal masses, black, web-like colonies on leaves, branches, and trunks of living trees	Conidia are small, hyaline, smooth-walled, unicellular, ellipsoid, cylindrical, and oval	Conidiophores short, poorly differentiated, hyaline	Pycnidia are superficial, large, dark brown or black. They have an elongated flask-shaped form with a protruding stoma at the apex
*Xenomorphomyces*	Developing on the surface of insects, (ants)	Conidial sporulation is hyaline, from round-oval to oval-elongated, often pear-shaped, unicellular, less commonly two- or three-celled	Conidiophores are solitary and may form clusters of conidia at the ends of the conidiophores. Branching conidiophores are observed	Pycnidia are absent. The formation of bed-like structures (clusters of conidiophores growing next to each other) can also be observed

## Data Availability

The data are available from the corresponding author upon reasonable request.

## Supplementary Materials

The following supporting information can be downloaded at: https://zenodo.org/records/21265283 (accessed on 10 August 2026). Video S1, video file of µCT cross-section and 3D reconstruction of fungal parts on the ant body.
